# Fertility Intention and Related Factors for Having a Second or Third Child Among Childbearing Couples in Shanghai, China

**DOI:** 10.3389/fpubh.2022.879672

**Published:** 2022-06-09

**Authors:** Chenfeng Zhu, Li Yan, Yang Wang, Sifan Ji, Yiqin Zhang, Jian Zhang

**Affiliations:** ^1^Department of Obstetrics and Gynecology, International Peace Maternity and Child Health Hospital, School of Medicine, Shanghai Jiao Tong University, Shanghai, China; ^2^Shanghai Key Laboratory Embryo Original Diseases, Shanghai, China; ^3^Shanghai Municipal Key Clinical Specialty, Shanghai, China

**Keywords:** fertility intention, second child, third child, childbearing age, influencing factors

## Abstract

**Background and Purpose:**

As the global fertility rate declines, China has issued two and three-child policies in the past 10 years. Therefore, this study serves to evaluate fertility intention rates and related factors in couples intending to have a second child and third child.

**Methods:**

A cross-sectional survey was conducted in mainland China from July to August 2021. Couples with one or two children were invited to participate in our study in order to collect information about more than one child fertility intention and the possibly related factors. Odds ratios (ORs) and their corresponding 95% confidence intervals (CIs) were calculated and adjusted for potential confounding factors.

**Results:**

Data was collected from a total of 1,026 couples. Among couples with one child, 130 (16.2%) couples had the intention to have a second child. Additionally, only 9.4% of couples with two children desired to have third child. The study revealed large differences in socioeconomic and personal factors between the two groups. For couples with intentions for a second-child, a female age >35 years (adjusted odds ratio, aOR 1.92), a first child's age range from 3 to 6 (aOR 3.12), annual child spending as a percentage of household income >30% (aOR 2.62), and children's educational barriers (aOR 1.55) were associated with lack of intent to have a second child. Similarly, among couples with two children, parents with family financial constraints (aOR 6.18) and children's educational barriers (aOR 4.93) are more likely to have lack of intent to have a third child. Here, we report that government policies encouraging fertility (aOR 0.04) can effectly promote couples to pursue a second or third child.

**Conclusion:**

Overall, couples with one or two children in Shanghai had a low intention to give birth to a second or third child. In order to increase the birth rates, it is necessary to implement policies to reduce the burden of raising children and provide relief to parent's pressure of rearing a child with increased free time.

## Introduction

Fertility has always been a key issue of national concern. The decline in fertility is becoming an inevitable trend in most countries around the world, especially in developed countries (1, 2). Low fertility rates are influenced by a range factor including medical infertility and lack of fertility intention (3). Couples with physical infertility may seek medical support (4). While often perceived as simply a behavioral choice, fertility intention needs to be clearly investigated regarding influencing factors such as sociodemographic and economic characteristics (5).

In the late 1970s, the one-child policy, which was also called “family planning,” was implemented to relieve the population pressure and support China in leaving severe poverty (6). During this policy, each couple was allowed to bear only one child. Now, several decades later, China has become one of the “low-fertility-rate countries” (7) and has had to meet new challenges which are aftereffects of the policy such accelerated population aging, skewed sex ratio, and decline in the working-age population, all of which are may hinder economic development (8).

**Figure 1:**
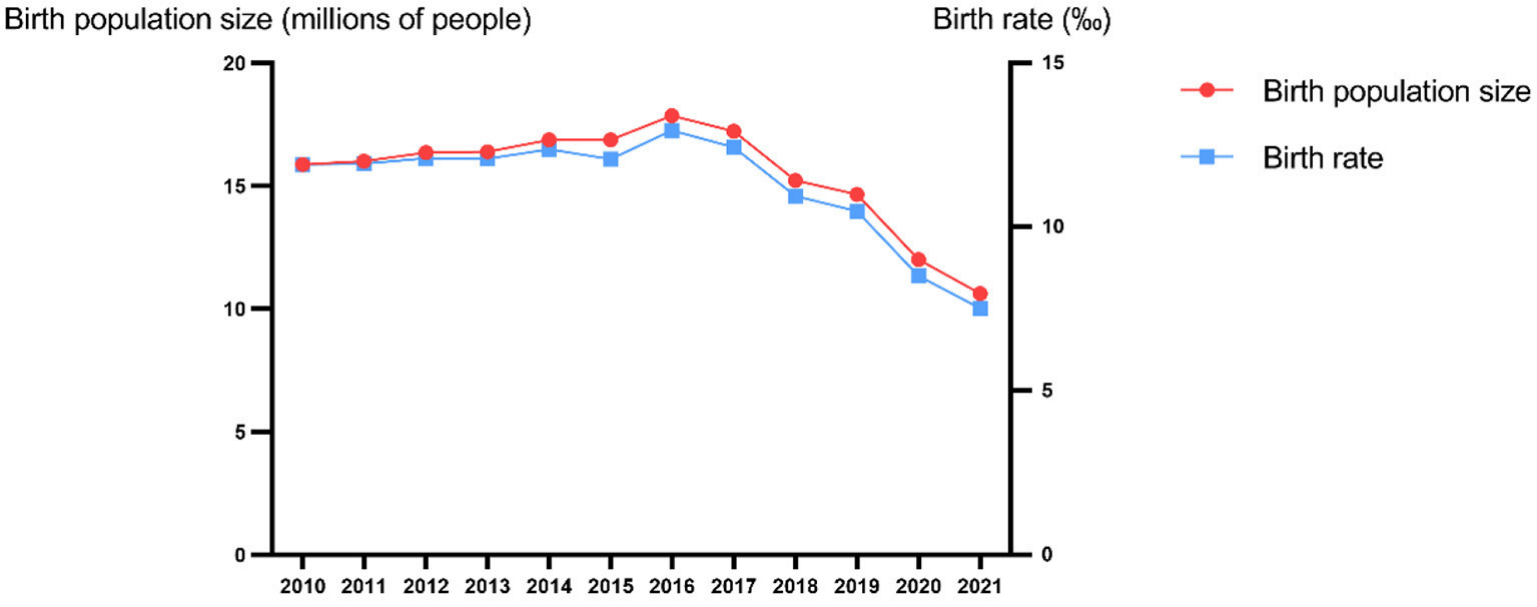
The Birth Population Size and Birth Rate in China.

Since 21st century, China has gradually changed its fertility policy, from a selective two-child policy to a comprehensive two-child policy. Nevertheless, according to the China Health Statistics Yearbook, the total annual births number in China has not shown an obvious growth trend (9) (Figure 1). On August 20, 2021, the Chinese government amended the Family Planning Law to allow a couple in China to have three children.

Recently, research on fertility intention has becomes the focus of fertility level because of the essential role of couples' behavior in the mediation of fertility outcomes (10). Although there are discrepancies between fertility intention and behavior at the individual level, on a population-level, fertility intention is correlated with having a child (11). Fertility intention is regarded to predict fertility behavior and it is affected by government policy as well as individual, social, economic, and cultural factors (12, 13). In China, previous studies have shown that the prevalence of the fertility intention was 39.4% for a second child in 2017, and 12.2% for a third child in 2021 among the general population (14, 15). According to this year's National Bureau of Statistics, 43% of the births in 2021 were second births. Therefore, it is imperative to evaluate the fertility intention for a second or third child and related factors. To date, few studies have been conducted to evaluate fertility intention in parous couples (16). Most studies of fertility intentions involve all couples of childbearing age. Here, we investigate the intention to have a second/third child among couples who already have one/two children to remove confounding factors and clearly understand mechanisms.

Considering remediating the current situation of low birth rates, efforts should be made to address associated factors in target families with one or two children. This study is designed to evaluate the factors influencing parous women's choice of have one more child. Thus, this research serves to provide information on the uptake of the universal multiple birth policy on individual, social, and economic levels so that a higher fertility rate may be achieved.

## Methods

### Study Design and Participants

We conducted a cross-sectional survey in Shanghai from July to August 2021. The study population was based on the female fertility prospective cohort established established between 2013 and 2017 at one of the pre-pregnancy centers in Shanghai, which contains information on basic sociodemographics and reproductive intentions (17). Couples with one or two children were invited to participate in our study to share information about their “second/third births intentions” and likely related factors.

The inclusion criteria of our study were as follows: females aged between 20 and 45 years old, married with one or two living children, no history of severe physical or mental diseases, and voluntary participation in the study. The private information of patients has been strictly protected and data has been anonymized. Patients had the right to refuse or quit the study at any point effective immediately.

### Definition of Fertility Intention

Fertility intention refers to people's desire to have children and pursuit of childbirth (18), which is impacted by the expectation of the number, timing, gender, and quality of the children. Fertility intention, which was the dependent variable and primary outcome, was measured by one question: “Do you intend to have another child?” with the response options being: “do not intend, intend, and uncertain” accordingly.

### Procedures

An in-person questionnaire and conversation were carried out to collect information from each participant. The questionnaire included the following components: sociodemographic characteristics of the women and their husbands (age, BMI, birthplace, education level, household annual income, family type, number of children, history of disorders, and pregnancy history, etc.), information of the first and second children in the family (age, gender, and economic investment, etc.), whether or not to have one more child and the reason related to it (whether to have second child for family with one child; whether to have third child for family with two child), ideal number of children (How many children do you think is appropriate in the family), and the factors which may influence the decision of having another child (economic status, working conditions, government policy, childcare, and challenges with access to children's education). The contents of the questionnaire were appropriately revised after review and pretest by professors in Gynecology (ZJ). Finally, the validity and reliability of questionnaire is established.

To ensure a high quality of data collection, the questionnaire was filled out by the investigator during the interview according to standard protocol. If participants did not answer any questions because they were unwilling to respond, it was treated that as missing information.

Couples with one child were divided into three groups: with second-child intention (those with fertility intent), without second-child intention (those who wanted no more children), and undecided (those who have not decided whether or not to have a second child). Accordingly, couples with two children were divided into three groups in the same way.

### Statistical Analysis

All of the data were analyzed using SPSS (Version 26.0, IBM, Corp, Armonk, NY, USA). Between the groups with and without second/third child intention, a chi-squared test was used to compare the differences in basic characteristics. A binary logistic regression with “intention for a second child” and “intention for a third child” as the dependent variable and demographic factors as explanatory variable was conducted to test the associations between the intention for a second or third child and demographic factors of the couples or their children. Univariable conditional logistic regression analysis was also used to calculate crude odds ratios (ORs) and their 95% confidence intervals (CIs). According to the univariate analysis results, the covariables with P <0.1 were included in the multivariate logistic regression model. Multivariate logistic regression analysis was performed to explore the potential factors and corresponding ORs. Statistical significance was indicated by *p*-value < 0.05.

## Results

### Basic Characteristics

**Figure 2:**
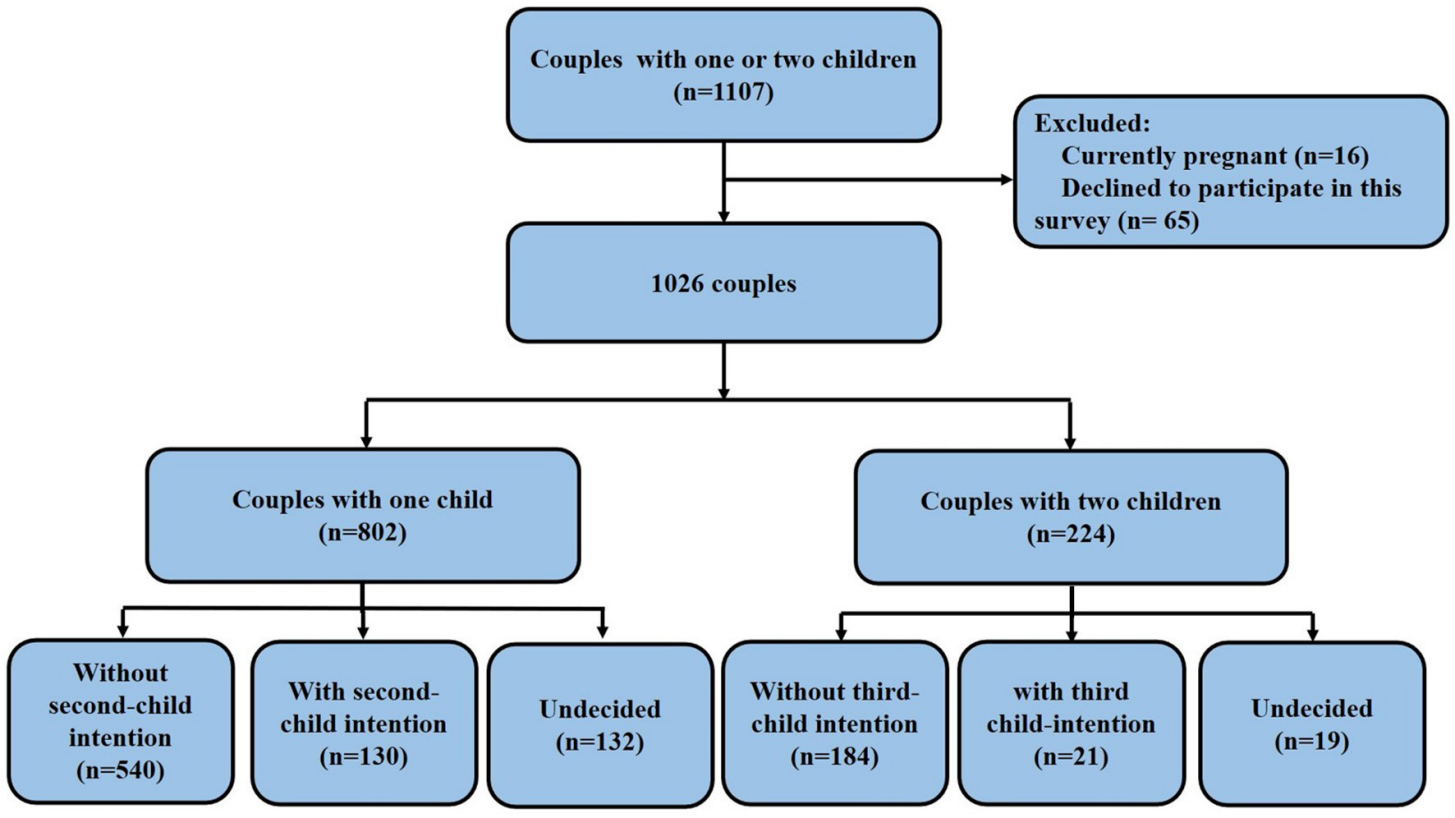
Flow chart.

Shown in Figure 2, a total of 1,107 women were recruited for the study and agreed to participate to the questionnaire in person. Then, 65 (5.8%) couples subsequently declined to participate in the survey and 16 (1.4%) couples were pregnant. As a result, 1,026 couples were enrolled in the study, including 802 (78.2%) couples with one child and 224 (21.8%) couples with two children.

Table 1 shows the sociodemographic characteristics of all participants. In the second-child intention group, the mean age [±standard deviation (SD)] was 34.91 (±3.40) for the women and 36.68 (±3.86) for the men. Furthermore, for couples in the third-child intention group, the female and male mean age [±(SD)] was 36.87 (±4.15) and 38.66 (±5.02). For most couples, their family's annual income was between 300,000 to 500,000 yuan. Couples with one child mostly came from only-child families, while families where only one parent was from an only child family accounted for most of the couples with two children.

**Table 1:** Sociodemographic characteristics of study participants.

	**ALL couples with one child**		**Have no second-child intention**		**Have second-child intention**		**Undecide**		***P*** ^**c**^	**ALL couples with second child**		**Have no third-child intention**		**Have third-child intention**		**Undecide**		***P*** ^**c**^
	***N** **=*** **802**^**a**^		***N** **=*** **540**		***N** **=*** **130**		***N** **=*** **132**			***N** **=*** **224**^**a**^		***N** **=*** **184**		***N** **=*** **21**		***N** **=*** **19**		
	***n***	**%**	***n***	**%**	***n***	**%**	***n***	**%**		***n***	**%**	***n***	**%**	***n***	**%**	***n***	**%**	
**Age , years , women**																
<35	432	53.87	267	49.44	85	65.38	80	60.61	0.001	76	34.08	62	33.88	8	38.10	6	31.58	0.814
≥35	370	46.13	273	50.56	45	34.62	52	39.39		147	65.92	121	66.12	13	61.90	13	68.42	
**Age , years , men**																
<35	304	41.30	186	37.27	53	47.75	65	51.59	<0.001	63	29.72	53	30.99	5	22.73	5	26.32	0.686
≥35	432	58.70	313	62.73	58	52.25	61	48.41		149	70.28	118	69.01	17	77.27	14	73.68	
**BMI** ^**b**^ **,women**																
<18.5	117	16.57	70	14.77	23	19.01	24	21.62	0.264	23	11.17	19	11.45	2	8.70	2	11.76	0.994
18.5-23.9	528	74.79	363	76.58	90	74.38	75	67.57		155	75.24	124	74.70	18	78.26	13	76.47	
≥24	61	8.64	41	8.65	8	6.61	12	10.81		28	13.59	23	13.86	3	13.04	2	11.76	
**BMI** ^**b**^ **,men**																
<18.5	16	2.36	11	2.42	3	2.56	2	1.87	0.619	2	0.98	2	1.22	0	0.00	0	0.00	0.700
18.5–23.9	417	61.41	272	59.78	79	67.52	66	61.68		128	62.75	106	64.63	12	52.17	10	58.82	
≥24	246	36.23	172	37.80	35	29.91	39	36.45		74	36.27	56	34.15	11	47.83	7	41.18	
**Education attainment, women**																
High school or low	42	5.61	23	4.61	11	9.02	8	6.30	0.395	21	9.91	14	8.14	4	18.18	3	16.67	0.366
Junior college or university	609	81.42	413	82.77	94	77.05	102	80.31		169	79.72	138	80.23	17	77.27	14	77.78	
Graduate or above	97	12.97	63	12.63	17	13.93	17	13.39		22	10.38	20	11.63	1	4.55	1	5.56	
**Education attainment, men**																
High school or low	40	5.37	21	4.22	13	10.74	6	4.76	0.028	20	9.43	12	6.98	4	18.18	4	22.22	0.088
Junior college or university	609	81.74	415	83.33	88	72.73	106	84.13		158	74.53	132	76.74	16	72.73	10	55.56	
Graduate or above	96	12.89	62	12.45	20	16.53	14	11.11		34	16.04	28	16.28	2	9.09	4	22.22	
**Birthplace, women**																
Shanghai	288	39.78	213	43.92	34	28.57	41	34.17	0.004	63	30.43	51	30.18	8	40.00	4	22.22	0.486
Other place	436	60.22	272	56.08	85	71.43	79	65.83		144	69.57	118	69.82	12	60.00	14	77.78	
**Birthplace, men**																
Shanghai	334	46.84	246	51.36	45	38.79	43	36.44	0.002	62	30.54	48	29.27	8	38.10	6	33.33	0.685
Other place	379	53.16	233	48.64	71	61.21	75	63.56		141	69.46	116	70.73	13	61.90	12	66.67	
**Annual household incomes (RMB)**																
<200,000	46	5.84	34	6.44	7	5.47	5	3.79	0.288	18	8.18	18	10.00	0	0.00	0	0.00	0.002
200,000–300,000	109	13.83	64	12.12	22	17.19	23	17.42		34	15.45	32	17.78	1	4.76	1	5.26	
300,000–400,000	212	26.90	144	27.27	36	28.13	32	24.24		56	25.45	47	26.11	1	4.76	8	42.11	
400,000–500,000	354	44.92	242	45.83	48	37.50	64	48.48		77	35.00	57	31.67	11	52.38	9	47.37	
>500,000	67	8.50	44	8.33	15	11.72	8	6.06		35	15.91	26	14.44	8	38.10	1	5.26	
**Parent's family situation**																
Both only child	389	48.50	271	50.19	54	41.54	64	48.48	0.237	59	25.99	46	25.00	7	29.17	6	31.58	0.844
Both non-only child	204	25.44	139	25.74	32	24.62	33	25.00		56	24.67	48	26.09	4	16.67	4	21.05	
One of only child	209	26.06	130	24.07	44	33.85	35	26.52		112	49.34	90	48.91	13	54.17	9	47.37	
**First child's age, years**																
≤ 2	157	19.58	90	16.67	32	24.62	35	26.52	0.013	8	3.52	7	3.80	1	4.17	0	0.00	0.746
3–6	621	77.43	436	80.74	91	70.00	94	71.21		106	46.70	88	47.83	11	45.83	7	36.84	
≥7	24	2.99	14	2.59	7	5.38	3	2.27		113	49.78	89	48.37	12	50.00	12	63.16	
**Second child's age, years**																
≤ 2	/		/		/		/		/	94	38.37	77	41.85	12	28.57	5	26.32	0.006
3–6										150	61.22	107	58.15	30	71.43	13	68.42	
≥7										1	0.41	0	0.00	0	0.00	1	5.26	
**Previous history of gynecological diseases**																
No	672	83.79	469	86.85	107	82.31	96	72.73	<0.001	198	88.79	158	87.78	23	95.83	17	89.47	0.499
Yes	130	16.21	71	13.15	23	17.69	36	27.27		25	11.21	22	12.22	1	4.17	2	10.53	
**Previous history of gynecological surgery**																
No	735	92.57	490	92.11	116	89.23	129	97.73	0.025	214	95.96	172	95.56	24	100.00	18	94.74	0.56
Yes	59	7.43	42	7.89	14	10.77	3	2.27		9	4.04	8	4.44	0	0.00	1	5.26	
**The item parents pay most for first child**																
Education	538	67.08	373	69.07	85	65.38	80	60.61	0.161	197	87.95	162	88.04	19	90.48	16	84.21	0.828
Others	264	32.92	167	30.93	45	34.62	52	39.39		27	12.05	22	11.96	2	9.52	3	15.79	
**The item parents pay most for second child**																
Education	/		/		/		/		/	79	35.27	61	33.15	9	42.86	9	47.37	0.348
Others										145	64.73	123	66.85	12	57.14	10	52.63	
**Annual first child expense** ^**d**^																
<70,000	579	72.56	370	68.90	105	81.40	104	78.79	0.001	104	46.85	87	47.80	8	38.10	9	47.37	0.002
70,000-100,000	148	18.55	118	21.97	15	11.63	15	11.36		68	30.63	62	34.07	2	9.52	4	21.05	
100,000–140,000	38	4.76	32	5.96	3	2.33	3	2.27		24	10.81	17	9.34	3	14.29	4	21.05	
>140,000	33	4.14	17	3.17	6	4.65	10	7.58		26	11.71	16	8.79	8	38.10	2	10.53	
**Annual second child expense**																
<70,000	/		/		/		/		/	197	88.74	165	90.66	16	76.19	16	84.21	0.134
70,000–100,000										17	7.66	13	7.14	2	9.52	2	10.53	
100,000–140,000										3	1.35	2	1.10	1	4.76	0	0.00	
>140,000										5	2.25	2	1.10	2	9.52	1	5.26	
**Factors affecting the fertility intention**																
**Family financial constraints** ^**e**^																
No	381	47.74	286	53.36	63	48.46	32	24.24	<0.001	76	33.93	57	30.98	15	71.43	4	21.05	<0.001
Yes	417	52.26	250	46.64	67	51.54	100	75.76		148	66.07	127	69.02	6	28.57	15	78.95	
**Working conditions** ^**f**^																
No	719	90.10	487	90.86	110	84.62	122	92.42	0.063	197	87.95	162	88.04	17	80.95	18	94.74	0.407
Yes	79	9.90	49	9.14	20	15.38	10	7.58		27	12.05	22	11.96	4	19.05	1	5.26	
**Fertility-encouraged government policy** ^**g**^																
No	655	82.08	448	83.58	97	74.62	110	83.33	0.053	149	66.52	135	73.37	2	9.52	12	63.16	<0.001
Yes	143	17.92	88	16.42	33	25.38	22	16.67		75	33.48	49	26.63	19	90.48	7	36.84	
**Childcare barriers** ^**h**^																
No	89	11.15	65	12.13	18	13.85	6	4.55	0.026	30	13.39	26	14.13	3	14.29	1	5.26	0.553
Yes	709	88.85	471	87.87	112	86.15	126	95.45		194	86.61	158	85.87	18	85.71	18	94.74	
**Children's educational barriers** ^**i**^																
No	279	34.96	172	32.09	60	46.15	47	35.61	0.010	100	44.64	77	41.85	15	71.43	8	42.11	0.035
Yes	519	65.04	364	67.91	70	53.85	85	64.39		124	55.36	107	58.15	6	28.57	11	57.89	

^a^*The sum does not necessarily equal the sample size for all variables because of missing data*.

^b^*BMI is defined as Body mass index. Body mass index is defined as weight in kilograms divided by the square of height in meters*.

^c^*Pearson's χ^2^ test*.

^d^*Annual first child expense: the total annual investment in children's life, education, etc*.

^e^*Family financial constraints: the shortage of money in raising more children*.

^f^*Working conditions: no extra time to raise more children due to work*.

^g^*Fertility-encouraged government policy: A policy to encourage childbirth by providing maternity subsidies and maternity leave*.

^h^*Childcare barriers: the shortage of time or labour in raising more children*.

^i^*Children's educational barriers: the stress of meet the educational needs of children*.

### Intention to Have a Second or Third Child

As shown in Table 2, among couples with one child, 130 (16.2%) couples had the intention to have a second child, while 540 (67.3%) couples had no intention of having another child, and another 132 (16.4%) couples had not yet decided. The majority of couples with two children (82.4%) showed no desire to have third child and only 9.4% of couples expressed the intention to have a third child. Overall, participants indicated that the ideal number of children was 1.72 ± 0.52. Furthermore, the ideal number of children among couples with one child was 1.61 ± 0.52. In contrast, the ideal number of children for couples with two kids was 2.06 ± 0.34.

**Table 2:** The overall intention for the number of children among study participants.

	***n***	**%**
**The number of ideal children among all couples (*****n*** **=** **1,026)**		
1	326	31.77
2	667	65.01
3	32	3.12
≥4	1	0.10
Mean ± SD	1.72 ± 0.52	
**The number of ideal children among couples with one child (*****n** **=*** **802)**		
1	322	40.15
2	468	58.35
3	12	1.50
≥4	0	0.00
Mean ± SD	1.61 ± 0.52	
**The number of ideal children among couples with two children (*****n** **=*** **224)**		
1	4	1.79
2	199	88.84
3	20	8.93
≥4	1	0.45
Mean ± SD	2.06 ± 0.34	
**Intention for a second child**		
No	540	67.33
Yes	130	16.21
Undecided	132	16.46
**Intention for a third child**		
No	184	82.14
Yes	21	9.38
Undecided	19	8.48

### Factors Influencing Fertility Intention

The crude odds ratios (cORs) and adjusted odds ratios (aORs) of factors associated with second-child intention are shown in Table 3. A female age >35 years [aOR 1.92 (1.27–2.91)] and annual child-related spending as a percentage of household income >30% [aOR 2.62 (1.05–6.53)] were associated with a lack of intent to have a second child. For couples with one child, education accounted for 67.1% of the total annual investment in that child. Families with two children spent 87% of child-related expenses on the first child's education. In the second child, the proportion of investment in education dropped to 35.3%. Educational barriers prevent parents from having a second child, regardless of being a one- or two-child families [one-child family: aOR 1.55(1.03–2.33); two-child family: aOR 4.93(1.34–18.14)]. Couples with a child aged 3 to 6 [aOR 3.12(1.19–8.17)] were less likely to have a second child. Moreover, the fertility-encouraged government policy [aOR 0.61(0.38–0.98)] makes it easier for couples to have a second child.

**Table 3:** Factors associated with second-child intention.

	**Have no second-child intention**	**Have second-child intention**	**Unadjusted OR (95% CI)**	***P***	**Adjusted OR (95% CI)**	***P***
	***N** **=*** **540**	***N** **=*** **130**	**No second-child intention vs. have second-child intention**		**No second-child intention vs. have second-child intention**	
**Age, years, women**						
<35	267	85	Ref	0.002	Ref	0.002
≥35	273	45	1.86 (1.27–2.77)		1.92 (1.27–2.91)	
**First child's age, years**						
≤ 2	84	30	1.41 (0.52–3.80)	0.023	1.97 (0.70–5.52)	0.017
3–6	371	90	2.40 (0.94–6.10)		3.12 (1.19–8.17)	
≥7	56	7	Ref		Ref	
**The percentage of child-related expense (%)** ^**a**^						
<10	370	105	Ref	0.054	Ref	0.117
10–30	118	15	1.47 (0.91–2.37)		1.26 (0.77–2.07)	
≥30	32	3	2.86 (1.17–6.95)		2.62 (1.05–6.53)	
**Fertility-encouraged government policy**						
No	448	97	Ref	0.053	Ref	0.039
Yes	88	33	0.58 (0.37–0.91)		0.61 (0.38–0.98)	
**Children's educational barriers**						
No	172	60	Ref	0.003	Ref	0.038
Yes	364	70	1.81 (1.23–2.68)		1.55 (1.03–2.33)	

^a^*The percentage of child expense (%) = 100% × Annual child-related expense/Annual household income*.

*The sum does not necessarily equal the sample size for all variables because of missing data*.

*Pearson's χ2 test*.

However, for families with two children, the age of the both of the two children and the financial investment in the second child have no effect on the intent to have the third child (Table 4). Among couples with two children, parents with family financial constraints [aOR 6.18(1.80–21.22)] are more likely lack intent to have a third child. Therefore, the government policy encouraging fertility may make people more willing to have a third child [aOR 0.03(0.01–0.17)].

**Table 4:** Factors associated with third-child intention.

	**Have no third-child intention**	**Have third-child intention**	**Unadjusted OR (95% CI)**	***P***	**Adjusted OR (95% CI)**	***P***
	***N** **=*** **184**	***N** **=*** **21**	**No third-child intention vs. have third-child intention**		**No third-child intention vs. have third-child intention**	
**Age, years, women**						
<35	62	8	Ref	0.700	Ref	0.713
≥35	121	13	1.20 (0.47–3.05)		1.24 (0.39–3.98)	
**First child's age, years**						
≤ 2	7	1	0.99 (0.41–2.40)	0.992	0.86 (0.21–3.46)	0.837
3–6	88	11	0.87 (0.10–7.71)		0.62 (0.05–8.63)	
≥7	89	12	Ref		Ref	
**The percentage of children expense (%)** ^**a**^						
<10	24	5	Ref	0.067	Ref	0.080
10–30	63	11	1.19 (0.38–3.89)		0.54 (0.12–2.53)	
≥30	93	5	3.88 (1.04–14.48)		4.34 (0.64–17.54)	
**Family financial constraints**						
No	57	15	Ref	<0.001	Ref	0.004
Yes	127	6	5.57 (2.06–15.10)		6.18 (1.80–21.22)	
**Fertility-encouraged government policy**						
No	135	2	Ref	<0.001	Ref	<0.001
Yes	49	19	0.04 (0.01–0.17)		0.04 (0.01–0.19)	
**Children's educational barriers**						
No	162	19	Ref	0.014	Ref	0.016
Yes	22	2	3.47 (1.29–9.36)		4.93 (1.34–18.14)	

^a^*The percentage of children expense (%) = 100% × Annual two children expense/Annual household income*.

*The sum does not necessarily equal the sample size for all variables because of missing data*.

*Pearson's χ2 test*.

## Discussion

In the context of an overall low fertility rate, the intention rates investigated here for second and third births were 16.2% and 9.4% in Shanghai, respectively. This finding is lower than that of Jue Liu who found the second-child intent rate was 39.4% in central and eastern China after the universal two-child policy (15). Likewise, the third-child intention rate in our survey was also lower than the data obtained nationwide by another scholar in 2021 which was 12.2% (14). Unlike previous studies (10), here, we conducted a cross-sectional survey among couples with one child for second-child intention and couples with two children for third-child intention, controls for confounding factors and provides more detail and therefore accuracy (19, 20). In addition, we found that the baseline age of the participants in this study was older than that of the previous two studies. We believe that this is related to the social culture of late marriage and late childbearing of Women in Shanghai, which is based on the rapid economic development of Shanghai (9). It can be observed that differences in race, region, and social culture lead to differences in fertility intention. A study from Bangladesh suggested that the willingness of local women to have a second child was 71.2%, and the willingness to have a third child was 55% (21). In Iran, a country also with a low fertility rate, 43% of families in the city of Tehran who already had a one child have a second child (22). In most developed countries, according to the National Survey of Family Growth (NSFG), about 12.8% of families were unwilling to have any children in 2017, and this trend increases each year (1).

Couples' age was one of the most important associated factors with the second-child intention. We found that women who were 35 and older were less likely to want to have a second child. Perhaps people realize that being an older woman can lead to infertility and more pregnancy complications (18). A large number of studies have confirmed that before the age of 35 is the optimal reproductive age for women, which is consistent with our results (23). However, this pattern was not reflected in the third-child intention population: women with two children were not influenced by their age in their intent to have a third child. A possible explanation is that the baseline age of women who wish to have a third child is older (mean age: 36.87), and women older than 35 account for 65.8% of the total “third child intent” population. In addition, older women are less likely to have children (12, 13). Most women are facing infertility troubles caused by advanced age, rather than other factors affecting their desire to have a third child.

Most studies have shown that the high cost of education can be a deterrent to fertility (15). Although most of the children attend tuition-free schools in Shanghai, many parents take additional tutoring or extracurricular education, which increases the cost. This study also confirms that the family financial burden of education in Shanghai is significantly negatively correlated with fertility willingness.

Children dependency ratio is the population of children aged 0–14 divided by the working population aged 15–64. According to the *China Statistical Yearbook-2021*, Shanghai has the highest per capita disposable income in China (72,232 RMB), while its children dependency ratios were the lowest in the country. The children dependency ratio of 13.25 in Shanghai is almost half of the national average of 26.24. The decline in the number of children a family needs to support leads families to pursue meritocratic individual education, where the cost of educating a single child is relatively high. Existing data confirms this; the per capita expenditure on education and culture of Shanghai residents ranks first in China (as shown in Supplementary Table 2). When the number of children in a family increase, the financial investment in education of the whole family will increase greatly without culturally changing the previous education model. This increased burden of education discourages couples from having a second or third child.

In another survey (13, 24), a family's socioeconomic status was found to be associated with raising a third child. Our findings confirm these results. In our study, family financial constraints have a negative effect on the third-child intention. Greater economic resources mean that families can afford the high costs of raising and educating children. This is also in accordance with additional observations in this study, such as couples whose annual child-related spending was >30% of annual were less likely to have a third child.

Furthermore, our results showed that couples had lower intentions to have a second child when the first child was 3–6 years old. A possible explanation for this might be that Chinese law requires children to start school after the age of 6, which provides couples more time and energy to consider raising another child. Consistent with the present results, previous studies have demonstrated that more disposable time increases a woman's desire to have children (13).

The decline in fertility and changes in population structure can facilitate the adjustment of national fertility policies (25). The new policies not only provide support and resources for couples, but also provide financial help in terms of medical insurance (26). On August 20, 2021, the implementation of China's three-child policy legally stipulates that couples can have three children and enjoy these supporting measures (27). For couples who wish to have third child, maternity leave, maternity benefits, and maternity insurance provide the free time and financial support which according to this study are key to choosing to have another child. Therefore, these factors may explain the relatively positive correlation between government policy encouraging fertility and greater fertility intention.

The fertility intention metric includes the ideal number of children, gender, and time to have children. In the absence of coercive birth restrictions, the number of children people have depended largely on how many they want. Some scholars have found that although China's population policy is gradually opening up, the ideal number of children has been declining in recent years, and economic development and the improvement of education level may likely contribute to a continuous decline in the ideal number of children (28). A couple's ideal number of children is affected by factors such as population policy, economic factors, and children's education, leading to a large gap between the ideal and reality. The gap is more pronounced among families with one child, rather than two. Most families with one child have an ideal number of two children, but economic, educational and other factors prevent them from pursuing a second child. The ideal number of children for families with a second child is close to 2, which is exactly what they expect. Therefore, in order to increase the levels of fertility intention, and thus increase the fertility level and birth rate, We must urge the government to introduce policies, such as providing maternity subsidies, or reducing the burden of children's education.

There are some limitations to our study. First, as this is a single-center study in Shanghai, the sample size is relatively small. Although our sample size still meets the basic statistical requirements and can detect a moderate effect size as significant, these results may not be applicable to other regions. In addition, the target population of this survey is people of childbearing age who have lived in Shanghai for a long time. In Shanghai, there are more people with higher education levels, higher incomes, and advanced ages, which has certain deviations from the national average. People in Shanghai cannot represent women in all regions of China. Future research may focus on people from different regions of China and different economic and cultural backgrounds. In addition, this cross-sectional study can accommodate the multiple child-bearing intentions of couples of childbearing age in Shanghai, providing valuable data for policy departments, but cannot determine causal relationships from these associations.

## Conclusion

In conclusion, couples with one or two children in Shanghai have low intentions to give birth to a second or third child. Their decision is probably influenced by female age, age of the first child, family economic conditions, children's education expenses, and national fertility policies. It is necessary to take measures to reduce the financial burden of raising children and increase free time for couples to provide relief from parents' pressure of rearing a child. Governments and societies need increase efforts to support the intention to have a second or third child and thus increase the national fertility rate.

## Data Availability Statement

The raw data supporting the conclusions of this article will be made available by the authors, without undue reservation.

## Ethics Statement

The studies involving human participants were reviewed and approved by the Institutional Review Board of the International Peace Maternity and Children's Health Hospital. The patients/participants provided their written informed consent to participate in this study.

## Author Contributions

JZ was conceived of the study and participated in its design, as well as supervised the study, and critically revised the manuscript. CZ performed the investigation and wrote the manuscript. LY and YW contributed to collection. YZ and SJ participated in statistical analysis. All authors read and approved the final version of the manuscript.

## Funding

This work was supported by National Key Research and Development Program (Grant Number 2018YFC1002102) and Shanghai Municipal Key Clinical Specialty, Shanghai, China (Grant Number shslczdzk01802).

## Conflict of Interest

The authors declare that the research was conducted in the absence of any commercial or financial relationships that could be construed as a potential conflict of interest.

## Publisher's Note

All claims expressed in this article are solely those of the authors and do not necessarily represent those of their affiliated organizations, or those of the publisher, the editors and the reviewers. Any product that may be evaluated in this article, or claim that may be made by its manufacturer, is not guaranteed or endorsed by the publisher.
